# Shared extracellular vesicle miRNA profiles of matched ductal pancreatic adenocarcinoma organoids and blood plasma samples show the power of organoid technology

**DOI:** 10.1007/s00018-020-03703-8

**Published:** 2020-11-25

**Authors:** Anikó Zeöld, Gyöngyvér Orsolya Sándor, Anna Kiss, András Áron Soós, Tamás Tölgyes, Attila Bursics, Ákos Szűcs, László Harsányi, Ágnes Kittel, András Gézsi, Edit I. Buzás, Zoltán Wiener

**Affiliations:** 1 Department of Genetics, Cell and Immunobiology, Molecular Cancer Biology Research Group, Semmelweis University, Budapest, Hungary; 2 Uzsoki Hospital, Budapest, Hungary; 3 1st Department of Surgery, Semmelweis University, Budapest, Hungary; 4 Institute of Experimental Medicine, Eötvös Loránd Research Network, Budapest, Hungary; 5 ELKH-SE Immune-Proteogenomics Extracellular Vesicle Research Group, Semmelweis University, Budapest, Hungary; 6 Department of Measurement and Information Systems, University of Technology and Economics, Budapest, Hungary; 7 Department of Genetics, Cell and Immunobiology, Semmelweis University, Budapest, Hungary; 8 HCEMM Kft Extracellular Vesicle Research Group, Semmelweis University, Budapest, Hungary

**Keywords:** PDAC, Collagen, Extracellular matrix, Organoid, Exosome, miR-21, miR-195

## Abstract

Extracellular vesicles (EV) are considered as a promising diagnostic tool for pancreatic ductal adenocarcinoma (PDAC), a disease with a poor 5-year survival that has not improved in the past years. PDAC patient-derived 3D organoids maintain the intratumoral cellular heterogeneity, characteristic for the tumor in vivo.Thus, they represent an ideal in vitro model system to study human cancers. Here we show that the miRNA cargo of EVs from PDAC organoids largely differs among patients. However, we detected a common set of EV miRNAs that were present in matched organoids and blood plasma samples of individual patients. Importantly, the levels of EV miR-21 and miR-195 were higher in PDAC blood EV preparations than in healthy controls, albeit we found no difference compared to chronic pancreatitis (CP) samples. In addition, here we report that the accumulation of collagen I, a characteristic change in the extracellular matrix (ECM) in both CP and PDAC, largely increases EV release from pancreatic ductal organoids. This provides a possible explanation why both CP and PDAC patient-derived plasma samples have an elevated amount of CD63 + EVs. Collectively, we show that PDAC patient-derived organoids represent a highly relevant model to analyze the cargo of tumor cell-derived EVs. Furthermore, we provide evidence that not only driver mutations, but also changes in the ECM may critically modify EV release from pancreatic ductal cells.

**Electronic supplementary material:**

The online version of this article (10.1007/s00018-020-03703-8) contains supplementary material, which is available to authorized users.

## Introduction

Pancreatic ductal adenocarcinoma (PDAC) is one of the most dangerous cancers with a 5-year survival rate of less than 8%. Although novel promising targets have been identified [1], there is only a slow progress in the early diagnosis and treatment efficiency of this disease. Most PDAC tumors prevalently harbour mutations in *KRAS*, encoding a key protein in the signalling pathway of epidermal growth factor (EGF), and/or *TP53* that is central in DNA damage responses. However, a large number of oncogenic driver mutations in several other genes lead to large intratumoral genetic heterogeneity [2]. Reliable biomarkers for PDAC still need to be identified. miRNAs, among them several with known roles in cancer initiation and progression, are promising candidates. However, identifying the cancer-specific expression of circulating miRNAs is obscured by the inherent heterogeneity of miRNA populations in the blood.

**Fig. 1:**
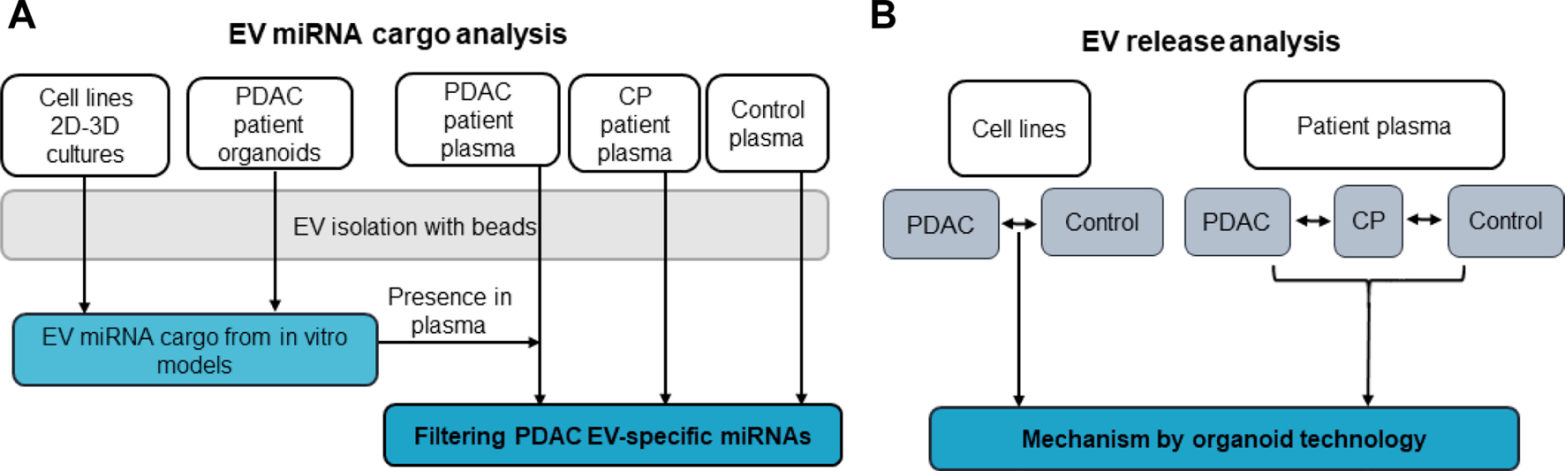
Experimental strategy to characterize EVs in PDAC. **a**–**b** Flow chart for determining PDAC-specific EV miRNAs (**a**) and to compare EV release (**b**)

Extracellular vesicles (EVs) are membrane surrounded structures with biologically active molecules, such as miRNAs, proteins, lipids and they transfer their cargo from the secreting to the target cells. Thus, EVs represent a novel intercellular communication mechanism, where many types of biologically active molecules are transmitted in a pre-packaged form. A specific subtype of secreted membrane vesicles, exosomes are characterized by a special biogenetic origin; they are formed from multivesicular bodies (MVB) [3]. Cancer exosomes have been shown to perform cell-independent miRNA biogenesis and they modulate functions in the recipient cells [4]. Transformed cells secrete exosomes in high amounts, thus, it has been proposed that the balance of MVB fate is shifted towards fusion with the cell membrane in the producing cancer cells, leading to exosomal release [5]. Importantly, since tumor-derived EVs transport their tumor-specific cargo in a protected and concentrated way in the body fluids, they hold a great promise for early tumor detection [6, 7]. Unfortunately, analyzing EV release and their cargo in the pancreas and in tumor tissues in vivo is difficult; furthermore, the potential use of EVs as biomarkers and understanding their in vivo roles in PDAC and in other pancreatic diseases is largely hindered by the lack of proper models. So far most studies focused on cell lines in two dimensional cultures in vitro, resulting in the large variability and conflicting results among model systems. The 3D organoid technology maintains the cellular heterogeneity that is characteristic for the original tissue, thus, it represents one of the most modern platforms to study human cancers [8, 9]. Importantly, organoids have been successfully isolated from PDAC patients and recently a PDAC organoid library has been established [10–13]. The most common traditional method of small EV isolation, including exosomes, is differential ultracentrifugation [14]. However, blood contains lipoproteins and other molecular complexes at a high quantity that may co-purify with EVs [15], leading to the detection of contaminating non-EV-associated molecules as well. Several reports have used this method when analyzing blood EV in PDAC and they led to a large variability among data. Thus, in order to detect EV release changes and EV miRNAs specific for PDAC, we applied here a combined approach using blood plasma samples, patient-derived organoids and an antibody-based EV isolation protocol (Fig. 1a, b). Interestingly, the miRNA profile of PDAC organoid-derived EVs overlapped with the matched patient-derived blood plasma EV cargo, showing the power of this technology. The EV concentration and miRNA profile, such as miR-21 and miR-195 levels differed between PDAC plasma EV and control samples; however, we found no change when comparing to chronic pancreatitis (CP) patients. Finally, we show that the accumulation of type I collagen in the extracellular matrix (ECM), a major hallmark of both CP and PDAC, critically contributes to the increase in EV release, providing evidence that EV secretion is modified not only by mutations, but also by the microenviroment already in a pre-tumorigenic stage.

## Materials and methods

### Cell culture

We used PDAC cell lines (derived from primary tumors) Panc02.03 (ATCC® CRL-2553), Panc08.13 (ATCC® CRL-2551), Panc10.05 (ATCC® CRL-2547) and Panc-1 (ATCC® CRL-1469). Panc02.03, Panc08.13 and Panc10.05 were cultured in RPMI-1640 (Gibco) supplemented with 15% FBS (Gibco), cyprofloxacine, antibiotic/antimycotic mix, glutamine (Sigma) and 10 U/ml human recombinant insulin (Santa-Cruz). Panc-1 cells were cultured in DMEM with 10% FBS (Gibco) and antibiotics. The control immortalized Human Pancreatic Duct Epithelial Cell Line (H6c7, HPDEC, Kerafast) was maintained in keratinocyte serum-free medium that contained EGF and bovine pituitary extract (Thermo Fisher, 17005042), antibiotic/antimycotic mix and glutamine (Sigma). Cells were tested for mycoplasma contamination with Hoechst staining and only negative cultures < 9 passage numbers were used in our experiments. Cells were washed three times with phosphate buffered saline (PBS) 2 days after culturing them in serum-free medium, the medium was then replaced and EVs were collected after 2 days. For 3D cultures, cells were removed from culture dishes (Eppendorf) with TrypLE (Gibco), embedded into Matrigel with 20,000 cells/droplet (25 μl) and cultured in 2.5% EV-free FBS (exosome depleted One-Shot FBS, Gibco) prior change to serum-free medium 2 days before starting EV collection.

### Human organoid and primary stromal cell cultures

The Medical Research Council of Hungary (ETT-TUKEB, https://ett.aeek.hu/en/secretariat/, No 51323-4/2015/EKU) as the national authority approved all experiments involving human samples and informed consent was obtained from the patients. Clinical data are provided in the Supplementary Information (Table S1). Tissue samples from patients undergoing operation were processed according to previously published methods [11]. The small cell clusters were embedded in Matrigel (25 μl droplets in 48 well plates (Eppendorf)) and cultured in pancreas organoid medium (POM) containing advanced DMEM/F12 with N2 and B27 supplements (Gibco), 10 mM HEPES (Sigma), 10 nM gastrin (Sigma), 1 mM N-Acetyl-Cysteine, glutamine, antibiotic/antimycotic mix (Sigma), 500 nM A83-01 (Sigma), 100 ng/ml murine Wnt3a (Peprotech), 500 ng/ml human R-Spondin1 (R&D Systems), 0.5% EV-free FBS (Gibco). In addition, the Rho kinase inhibitor Y27632 (10 µM, Sigma) was added after tissue isolation for 3 days to avoid anoikis [16]. Organoids were isolated from Matrigel in every 7–10 days by centrifugation at 300 g for 8 min, mechanically disrupted, washed with PBS and they were then embedded in Matrigel again. FBS was removed before collecting EVs.

For RNA isolation from organoid supernatant-derived EVs, 1 mL of the samples was centrifuged at 300 g for 5 min, 2000 g for 20 min and 12,500 g for 20 min, and the supernatants were incubated with 40 µL anti-CD63 and 20 µL anti-CD81-coated beads overnight (O/N). After washing with PBS, the beads with EVs were lysed with 700 μl Qiazol (Qiagen).

To prepare primary stromal cell cultures, attached cells after the first subcultivation of the 3D organoid cultures were trypsinized and cultured in DMEM high glucose + 5% FBS with antibiotics. Cells were used for experiments at 5–7 passages.

### Mouse organoid cultures

The Pest County Government Office of Hungary (the competent veterinary authority) approved the maintenance and experiments with mice. All animals were housed in IVC racks, on a cycle of 12L:12D and experiments were carried out according to and with the approval of the Institutional Animal Care and Use Committee (Semmelweis University). Animal experiments followed the principles of the Declaration of Helsinki. Pancreatic ducts were isolated from C57Bl/6 J mice (000664, The Jackson Laboratory) under microscope and embedded in Matrigel as described previously [17]. They were then cultured in mouse organoid medium (MOM): DMEM/F12 supplemented with 2% antibiotic/antimycotic mix, B27 supplement, gastrin (10 nM), 1.25 mM N-Acetyl-Cysteine, mouse R-Spondin1 (500 ng/mL, R&D Systems), murine noggin (100 ng/ml, Peprotech), nicotinamide (10 mM, Sigma), FGF-10 (100 ng/mL, Peprotech) and EGF (50 ng/ml, Peprotech). Organoids were removed from the 3D matrix in every 7–8 days, they were then centrifuged at 300 g for 5 min and mechanically splitted for further culturing. Cell clusters were then embedded into Matrigel droplets (25µL). In some experiments, organoids were treated with IL-1β (25 ng/mL), IL-6 (25 ng/mL) or TNFα (50 ng/mL) (all from Peprotech).

### Collagen-based organoid cultures

Mouse organoids were removed from Matrigel, and they were centrifuged at 300 g for 5 min. Next they were washed with PBS twice and embedded in collagen type I (Ibidi). Sixty µL water, 10 µL 10 × MEM (Gibco) and 30 µL collagen I were mixed for 100 µL collagen matrix on ice, and the pH was set to 7.2 with 1 M NaOH. In case of culturing in Matrigel/collagen mixtures, Matrigel and the prepared collagen solution were mixed at a ratio of 1:1. Organoids were then suspended in collagen or Matrigel/collagen mix and were cultured in 25 µL droplets in 48-well plates (Eppendorf) in MOM. Cells were removed from collagen with collagenase II (Sigma) applied for 30 min at 37 °C, and cells were then centrifuged at 300 g for 5 min.

### Tunable resistance pulse sensing (TRPS, qNano) and nanoparticle tracking analysis (NTA)

Culture supernatants were collected after 48 h, centrifuged at 300 g for 5 min and at 2000 g for 20 min to remove cells, cell debris and large EVs, respectively. To determine the size distribution and concentration of particles, supernatants were applied to TRPS (Izon) on membranes with a pore size of 400 nm (analysis range 185–1100 nm) with calibration beads CPC400G (Izon). Beads and samples were suspended and diluted in the same media. Minimum 500 data points were collected or samples were measured for 5 min.

Ten µL of cell or organoid culture supernatant (chemically defined medium without FBS) was diluted to 1 mL in PBS for NTA measurements. The particle size distribution and concentration were measured on a ZetaView Z-NTA instrument (Particle Metrix). According to the default settings, eleven cell positions were scanned at 25 °C for each sample to increase the reliability of the measurements. The camera settings applied were as follows: auto expose, gain: 28.8, offset: 0, shutter: 100, sensitivity: 80. The videos were analyzed with a minimum area of 5, maximum area of 1000 and a minimum brightness of 20 by the ZetaView Analyze software 8.05.10. The particle concentration measurements were normalized to cell number and to cell free medium control.

### Transmission electron microscopy (TEM)

The EV pellet was fixed within the ultracentrifugation tube with 4% PFA for 60 min. The sample was then post-fixed in 1% OsO_4_ for 15 min, rinsed with distilled water, block-stained with 1% uranyl acetate in 50% ethanol for 20 min, dehydrated in graded ethanol and embedded in Taab 812 (Aldermaston, T031). After polymerization at 60 °C O/N, 50–60 nm ultrathin sections were cut with a Leica UCT ultramicrotome (Leica Microsystems, UK). TEM images were taken with a Hitachi 7100 TEM instrument (Hitachi Ltd, Japan) equipped with a Veleta 2 k × 2 k MegaPixel side-mounted TEM CCD camera (Olympus, Tokio, Japan).

### EV detection by anti-CD63 or anti-CD81-coated beads

Culture supernatants were differentially centrifuged at 300 g for 5 min, 2000 g for 20 min and 12,500 g for 20 min. EVs from the supernatant were then bound to antibody-coated beads that had been blocked with 0.1% BSA (Sigma) in PBS for 30 min. Twenty µL of anti-CD63 coated beads (Thermo Fisher, 10606D) or 6 µL anti-CD81 coated beads (Thermo Fisher, 10616D) were added to 500 µL supernatant and incubated O/N with continuous rotation at 4 °C. Beads were magnetically separated, washed with PBS three times and they were labelled with anti-CD63-PE or anti-CD81-FITC (2 µL antibody/sample) for 20 min. Ten thousand beads were analyzed on a FACSCalibur (BD) instrument. Cells were counted with Burker chamber and results were normalized to cell number. The applied antibodies are listed in the Supplementary Information (Table S2).

### Mouse EV detection by anti-CD81-coated beads

Anti-CD81 antibody was bound to magnetic beads by the Dynabeads Antibody Coupling Kit (Invitrogen) according to the manufacturer’s protocol, using 10 µL antibody to 2 mg beads. One µL of the antibody-coated beads was then added to 250 µL supernatant. EVs captured by the beads were detected with PE-anti-CD81 antibody using a FACSCalibur instrument. The results were normalized to cell number.

### Whole-mount staining

Organoids were cultured in 4 or 8-well chamber slides (BD Biosciences), fixed in 4% PFA for 40 min, washed with PBS containing 4% NaCl, blocked and permeabilized in blocking buffer (5% FBS, 0.2% BSA, 0.3% Triton X-100 in PBS) for 60 min. Primary antibodies (Table S2) were applied O/N at + 4 °C in blocking buffer. After washing in PBS containing 0.3% Triton X-100 and 4% NaCl and incubating with the secondary antibodies in blocking buffer (Table S2), the samples were mounted in ProLong™ Diamond Antifade Mountant with DAPI (Thermo Fisher) and analyzed with a Zeiss LSM800 confocal microscope. Stromal cell cultures were treated with Brefeldin A (Sigma) for 24 h prior fixation with 4% PFA, double-stained with anti-IL-6, anti-αSMA and secondary antibodies and mounted in ProLong™ Diamond Antifade Mountant with DAPI.

### Human blood samples

The Medical Research Council of Hungary (ETT-TUKEB, No 13160-3/2017/EKU) approved all experiments involving human blood samples and informed consent was obtained from the patients (Table S1). Blood samples were collected in ACD-A tubes [18] (Greiner Bio-One, 455055), centrifuged twice at 2500 g for 15 min and the platelet free plasma (PFP) was aliquoted and stored at − 80 °C. When the amount of EVs was analyzed with the semi-quantitative bead-based method, PFP samples were differentially centrifuged (2000 g and 12,500 g for 20 min) and 500 µL samples were incubated with 20 µL anti-CD63-coated beads overnight with rotation at 4 °C. The percentage of positive beads was determined by anti-CD63 PE or anti-CD81 FITC antibodies and flow cytometry. When isolating RNA from blood-derived EVs, 1 mL of the sample was centrifuged at 300 g for 5 min, 2000 g for 20 min and 12,500 g for 20 min. The supernatants were incubated with 40 µL anti-CD63 and 20 µL anti-CD81-coated beads overnight. After washing with PBS, the beads with EVs were lysed in 700 μl Qiazol (Qiagen).

### EV isolation from PDAC cell culture supernatant

Two mL cell culture supernatant was centrifuged at 300 g for 5 min, 2000 g for 20 min and 12,500 g for 20 min and then splitted for comparison of the EV isolation methods. One mL of the centrifuged supernatant was ultracentrifuged at 100,000 g for 70 min. As a washing step, the pellets were resuspended in PBS and ultracentrifuged again. EVs were then lyzed in 700 µL Qiazol (Qiagen) for total RNA isolation. To compare the purity of EV preparates from different methods, we used antibody-coated beads as well. Fourty µL anti-CD63 and 20 µl anti-CD81-coated beads were added to 1 mL centrifuged supernatant and after O/N incubation and washing with PBS, the beads coated with EVs were lyzed in 700 µl Qiazol (Qiagen).

### mRNA and miRNA analysis

For mRNA analyis, organoid or cell-derived RNA was isolated with the RNEasy Micro Kit (Qiagen) according to the manufacturer’s protocol. RNA concentration was determined with NanoDrop. Half µg RNA was reverse transcribed with the SensiFAST™ cDNA Synthesis Kit (Bioline) and quantitative PCR reactions using TaqMan assays were carried out using the SensiFAST™ Probe Hi-ROX Kit (Bioline) on an ABI 7900HT Fast real-time PCR instrument (5 µL final reaction volume, 384-well format). The assays are listed in the Supplementary Information (Table S3).

When analyzing miRNAs, total RNA, including miRNA, was isolated with the miRNEasy Micro Kit (Qiagen) following the manufacturer’s description from the same initial sample amounts or cell numbers. For measuring individual miRNAs, 2 µL total RNA was reverse transcribed with the TaqMan® Advanced miRNA cDNA Synthesis Kit (Thermo Fisher) according to the manufacturer’s protocol. miRNA levels were then analyzed with the TaqMan® Fast Advanced Master Mix, TaqMan® Advanced miRNA Assays (Thermo Fisher) and an ABI 7900HT Fast real-time PCR instrument with 42 cycles. The assay IDs are listed in Table S3.

For miRNA array cards, 3 µL total RNA was reverse transcribed with Megaplex RT primers, the samples were pre-amplified with Megaplex PreAmp Primers (Thermo Fisher) and the TaqMan™ Array Human MicroRNA A Cards v2.0 (Thermo Fisher) were measured on an ABI 7900HT instrument according to the manufacturer’s description. The threshold was set to 0.2 and Ct < 42 was regarded as”miRNA present”. GeNorm (https://genorm.cmgg.be/) and NormFinder (https://moma.dk/normfinder-software) algorithms were used to calculate the gene expression stability for each potential reference miRNA, based on the average pairwise variation between all candidate reference miRNAs. NormFinder algorithm also takes into consideration the intra- and inter-group variability. Based on these analyses, miR-19b was selected for normalization, and the ΔCt value was calculated for each miRNA according to the following formula: ΔCt = Ct(miR-19b) – Ct(miR of interest). Before any further analysis, we removed miRNAs that were present in the control samples that contained no cells. In case of miRNA cargo analysis of cell line-derived EVs, only miRNAs that were detected in both replicates of the same condition were used. When comparing two conditions, miRNAs present in all samples of a specific comparison were applied for correlation analysis. For evaluating plasma-derived data, Ct values were normalized and a Ct value of 42 was used for miRNAs with an undetermined flag to obtain numerical data for statistical tests that is a widely accepted analysis approach.

### Sequencing

RNA from organoids was reverse transcribed with the SensiFAST™ cDNA Synthesis Kit (Bioline) and cDNA was amplified with Phusion High Fidelity DNA Polymerase (Thermo Fisher), using the following primers: *TP53*: TGAAGCTCCCAGAATGCCAG and CTTCAGGTGGCTGGAGTGAG (65 °C), *TP53* (DNA binding domain): CCCTGCCCTCAACAAGATGT and CTCAAAGCTGTTCCGTCCCA; *KRAS*: CCCAGGTGCGGGAGAGA and AACAGTCTGCATGGAGCAGG (65 °C). PCR products were then isolated from 2% gel, purified by the Gel Purification Kit (Macherey–Nagel) and sequenced by the forward primers with an Applied Biosystems 3500 Genetic Analyzer instrument (Life Technologies). Results were analyzed by Chromas 2.6 software (Technelysium Pty Ltd).

### Statistical analysis

Student’s unpaired or paired *t* test, one-way ANOVA and Tukey post hoc test, Kruskal–Wallis test with Dunn post hoc test were used with *p < 0.05, **p < 0.01 and ***p < 0.005 significance levels. Statistical differential expression of miRNAs was determined by the R statistical software (R Foundation for Statistical Computing, Vienna, Austria; version 3.5.1) and the limma package [19]. The Benjamini–Hochberg method was used for multiple testing correction when analyzing miRNA array data. Genes were considered to be differentially expressed when the adjusted *p* value false discovery rate was below 0.05. Microsoft Excel, SPSS version 25, R and Sigma Plot softwares were used for statistical evaluation and visualization.

## Results

### Changes in the miRNA cargo of PDAC EVs depend on the culture conditions

**Fig. 2:**
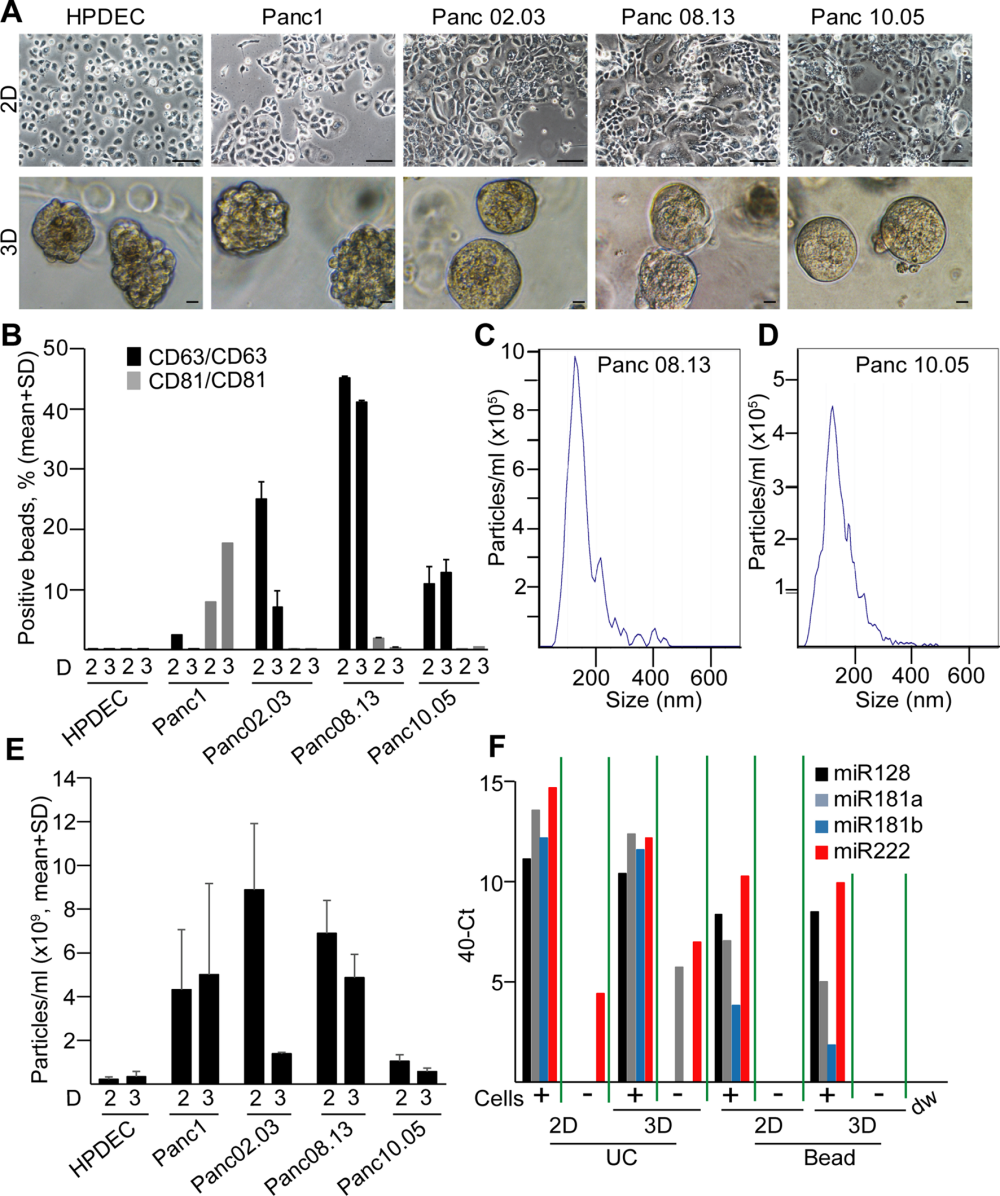
PDAC cell lines produce more EVs than control pancreas ductal cells both in 2D and 3D cultures. **a** The morphology of different cell lines in 2D and their colonies in 3D. Note that HPDEC is an immortalized pancreatic ductal control cell line. **b** CD63 + /CD81 + extracellular vesicle release of the indicated cell lines, measured by antibody-coated beads and flow cytometry (normalized to 10^6^ cells). The numbers after ‘D’ mean 2D or 3D cultures. **c**–**d** The distribution of particle size from 3D cultures of PDAC cell lines, determined by NTA. Note that cells, cell debris and large EVs were removed by serial centrifugation at 300 g and 2000 g. **e** Quantification of the particle concentration from 2 and 3D cultures (*n* = 4, data are normalized to 10^6^ cells). **f** miRNA levels in EV preparates isolated from Panc10.05 cells with serial centrifugation and ultracentrifugation (UC) or by a mixture of anti-CD81 and anti-CD63-coated beads (beads), measured by miRNA assays. Note that the empty, serum-free medium was used as control (Cells-). Scale bars: 50 µm (**a**, upper panels) or 10 µm (**a**, lower panels)

To characterize the miRNA cargo of PDAC cell-derived EVs, we first analyzed commercially available PDAC and control ductal cell lines. Traditional 2D cell cultures do not model the spatial organization of cells and tissues, which may influence the results when testing EV release and their molecular composition in PDAC. To develop a model system for studying PDAC-derived EV cargo, we set up 3D culture of cell lines in Matrigel, an often used extracellular matrix that is enriched for laminin and collagen type IV. Importantly, all the tested cell lines formed colonies in 3D after 1 week in culture, although with different colony morphologies (Fig. 2a). CD63 and CD81 are widely accepted markers of the small EV population. EVs can be assessed by a quick flow cytometry-based semi-quantitative method, using anti-CD63 or anti-CD81-coated beads that bind EVs [20, 21]. Interestingly, we observed a higher percentage of positive beads when using anti-CD63 as a detecting antibody compared to anti-CD81 in a PDAC cell line (Panc10.05) and this was independent of the capturing antibody (anti-CD63 or anti-CD81) (Fig. S1A), suggesting that these EVs carry CD63 at a higher amount than CD81. We detected positive beads with anti-CD63 in the pellet, but not in the supernatant that is EV free after ultracentrifugation (Fig. S1A). Importantly, ultracentrifugation is a commonly used method for small EV isolation. Triton X-100 had been shown to disrupt EVs, but not immune complexes [22]. As expected, Triton X-100 led to the drop in the percentage of positive beads both in cell-derived medium and in the ultracentrifuged pellet (Fig. S1B). Thus, the lack of positive beads in the supernatant after ultracentrifugation and the effect of Triton X-100 prove that when using anti-CD63 for both capturing and detection, we measured EVs with the bead method. Importantly, we detected EVs both in 2D and in 3D cultures, and PDAC cell lines produced more EVs compared to the control pancreatic ductal cell line (HPDEC) (Fig. 2b). To obtain comparable results, we normalized the percentage of positive beads to cell number and we used the same cell concentrations. The presence of particles in the supernatant of PDAC cell lines both in 2D and in 3D culture conditions was verified by Nanoparticle Tracking Analysis (NTA) as well (Fig. 2c–e).

**Fig. 3:**
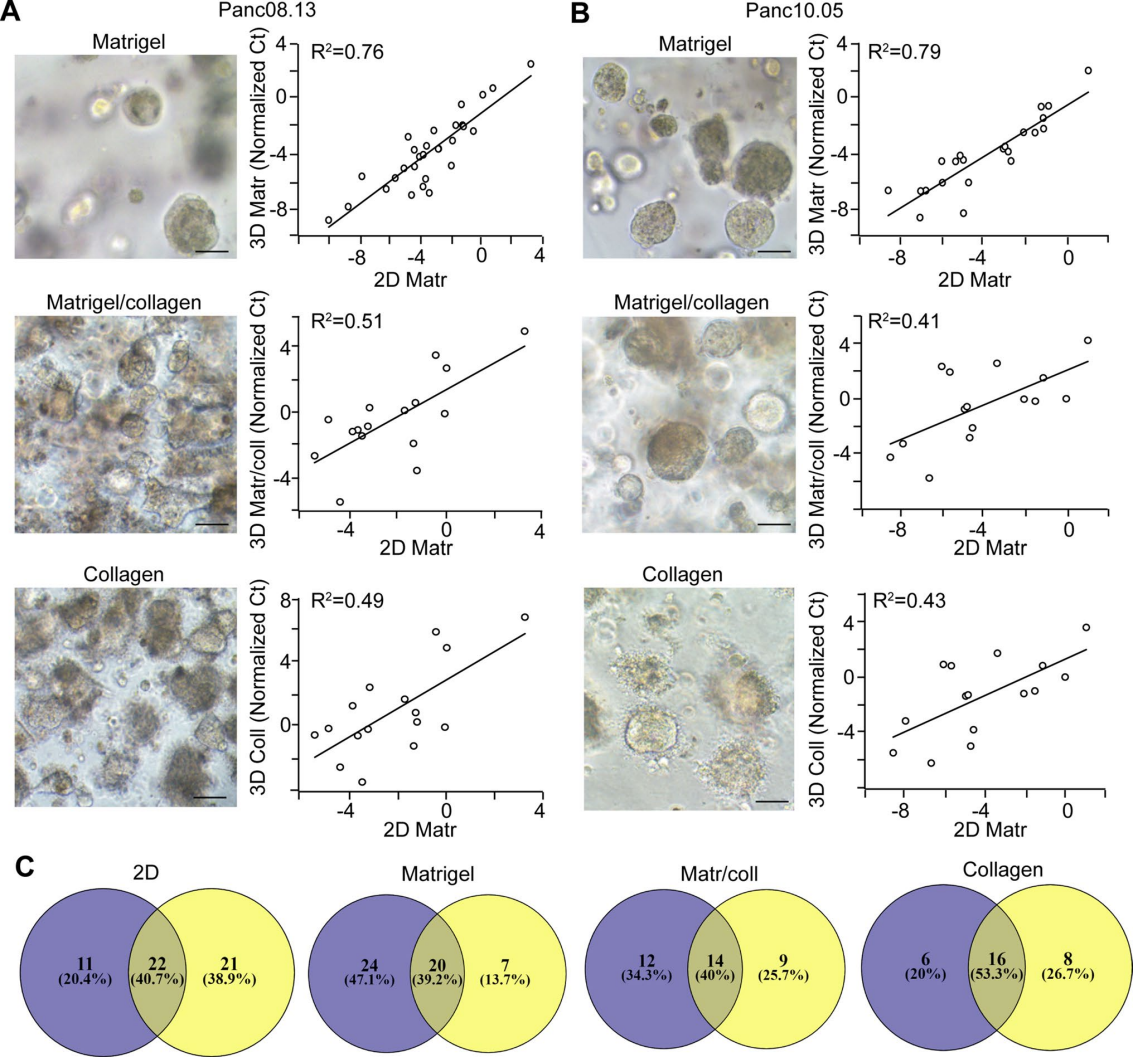
Effect of 3D culturing on the miRNA cargo of PDAC cell-derived EVs is dependent on the matrix. **a**–**b** The morphology of the colonies in different matrices (left panels) and correlations between the miRNA levels of EV samples from 2D and 3D cultures in the indicated matrices for Panc08.13 (**a**) and Panc10.05 (**b**) cells (low-density miRNA cards). Ct values were normalized to the level of miR-19b. **c** The number and percentage of the overlapping and non-overlapping miRNAs of Panc08.13 (blue color) and Panc10.05 (yellow circles) cell-derived EVs cultured under the indicated conditions. Scale bars: 50 µm (**a**, **b**)

We then compared the cargo of EVs isolated from 2 and 3D cultures by measuring the levels of 377 miRNAs from two PDAC cell lines with two replicates (Table S4). Since we found that the bead-based isolation of EVs led to a lower unspecific miRNA background compared to the widely used differential ultracentrifugation when studying some miRNAs whose presence had already been proved in EVs in another system (Fig. 2f and [21]), we selected this approach for our further screening experiments. For the analysis when normalization was required, we selected miRNAs with a stable level in EV preparations. Using two algorithms (Table 1), miR-19b and miR-484 were ranked among the top five hits in both lists. Since miR-484 was detected in some medium control samples as well, we chose miR-19b for normalization. We found a good correlation between the technical replicates for both cell lines in 2D and 3D in Matrigel (Fig. S2A–B), suggesting that the screen could be used to analyze the miRNA cargo of EV samples. Interestingly, when comparing miRNA screens of 2D and 3D cultures, we found a good correlation as well (Fig. 3a, b upper panels). Thus, these results indicate that this low-density array can be used for the analysis of EV cargo both in 2D and 3D cultures and Matrigel has only a modest effect on the EV miRNA cargo when analyzing 377 miRNAs.

**Table 1:** miRNAs with a stable level across PDAC cell line-derived samples with two different algorithms

Rank	Algorithms	
	geNorm	NormFinder
1	hsa.miR.16	**hsa.miR.19b**
2	**hsa.miR.19b**	hsa.miR.146a
3	hsa.miR.484	hsa.miR.24
4	hsa.miR.17	hsa.miR.484
5	hsa.miR.223	hsa.miR.92a

The miRNA selected for normalization is indicated in bold

To test other 3D matrices as well, we used a 50–50% mixture of Matrigel/collagen I and pure collagen I, another frequently used scaffolding matrix. Similar to the 2D and Matrigel cultures, the replicates of miRNA screens (normalized to miR-19b) resulted in a high correlation (Fig. S2A, B). However, this correlation dropped when comparing 2D and 3D conditions for both cell lines in either Matrigel/collagen I or in collagen I (Fig. 3a, b). This drop was accompanied with a morphological change of the colonies in the presence of collagen I (Fig. 3a, b). Furthermore, the low percentage (40–50%) of overlapping EV miRNAs of the two cell lines was similar in 2D cultures, in Matrigel, in Matrigel/collagen I and in collagen I (Fig. 3c). Collectively, the effects of 3D culturing depend on the matrix, and neither Matrigel nor collagen could reduce the large variation in the EV miRNA cargo that can be observed among cell lines.

### miRNA profile of PDAC organoid-derived EVs overlaps with patient derived blood plasma EV cargo

**Fig. 4:**
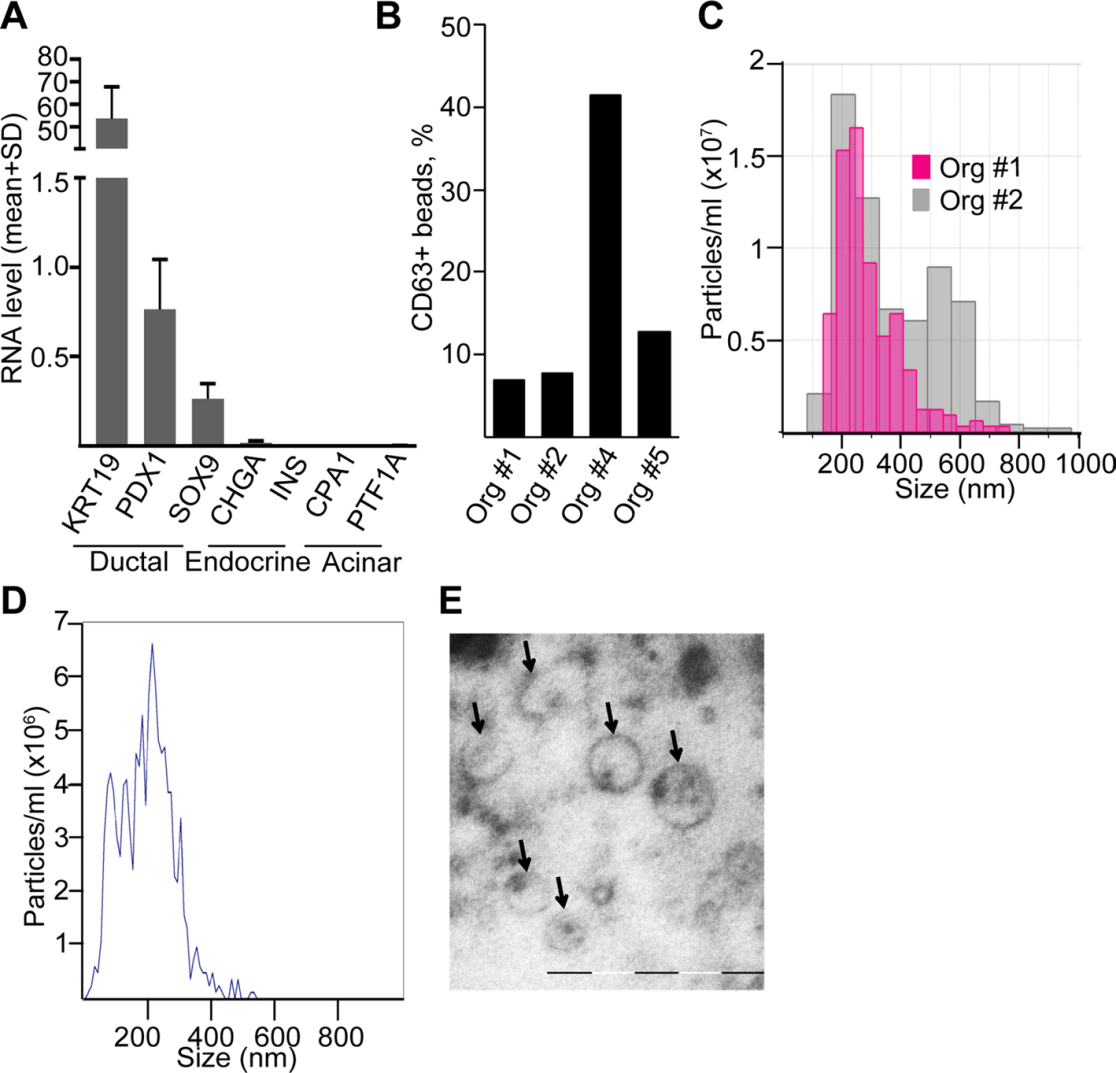
Human PDAC patient-derived organoids release EVs. **a** The RNA levels of ductal, acinar and endocrine markers in patient-derived organoids (*n* = 5, RT-qPCR). **b** Percentage of CD63 + beads from the supernatants of PDAC organoids, detected by flow cytometry. Note that all cultures released CD63 + EVs. **c** Detecting particles from organoid cultures with TRPS after centrifugation with 300 g and 2000 g. Membrane with pore size of 400 nm was applied (measuring range 185–1100 nm) and the calibration beads were used in the same medium. **d**–**e** Representative image of an NTA measurement (**d**) and transmission electron microscope (TEM) (**e**). Scale bar: 500 nm (**e**)

Studying tumor cell-derived EVs in vivo is technically challenging and there is a large variation among cell lines in their EV miRNA cargo in 3D as well. Thus, as a model that may better represent the in vivo situation, we next set up the 3D organoid culturing from PDAC patients. Organoids capture the cellular heterogeneity of in vivo tumors and thus, the organoid system is regarded as one of the best current methods to model human tumors. Since the presence of collagen I resulted in a morphological change of colonies derived from PDAC cell lines, we used Matrigel that is the standard matrix for the organoid technology. Organoids expressed the ductal markers *KRT19*, *PDX1* and *SOX9* and we could not detect the expression of the acinar markers *PTF1A* and *CPA1* or the endocrine pancreas markers *CHGA* and *INS* (Fig. 4a)**.** Importantly, all of our organoid lines could be maintained in culture without epidermal growth factor (EGF), proving that all of them have a mutant EGF receptor signaling pathway, although not all of them carried mutations in *KRAS*. Importantly, all the used cultures grew even in the absence of noggin, which is another hallmark of tumor organoids [10]. In addition, most of our PDAC organoid lines carried *TP53* mutations and they could be maintained in the presence of the Mdm2-inhibitor nutlin-3 that prevents the degradation of p53 (Table S5), leading to cell cycle arrest and ultimately to apoptosis only in cells with wild-type p53 [23]. Collectively, these data show that our cultures contain organoids of PDAC origin. We detected EVs in the supernatant of PDAC organoid lines using anti-CD63 beads and flow cytometry (Fig. 4b). Tunable resistive pulse sensing (TRPS) and NTA analyses confirmed the presence of particles in culture supernatants (Fig. 4c, d**).** Furthermore, transmission electron microscopic (TEM) images from ultracentrifuged culture supernatants proved the EV identity of the particles in the conditioned media (Fig. 4e).

**Fig. 5:**
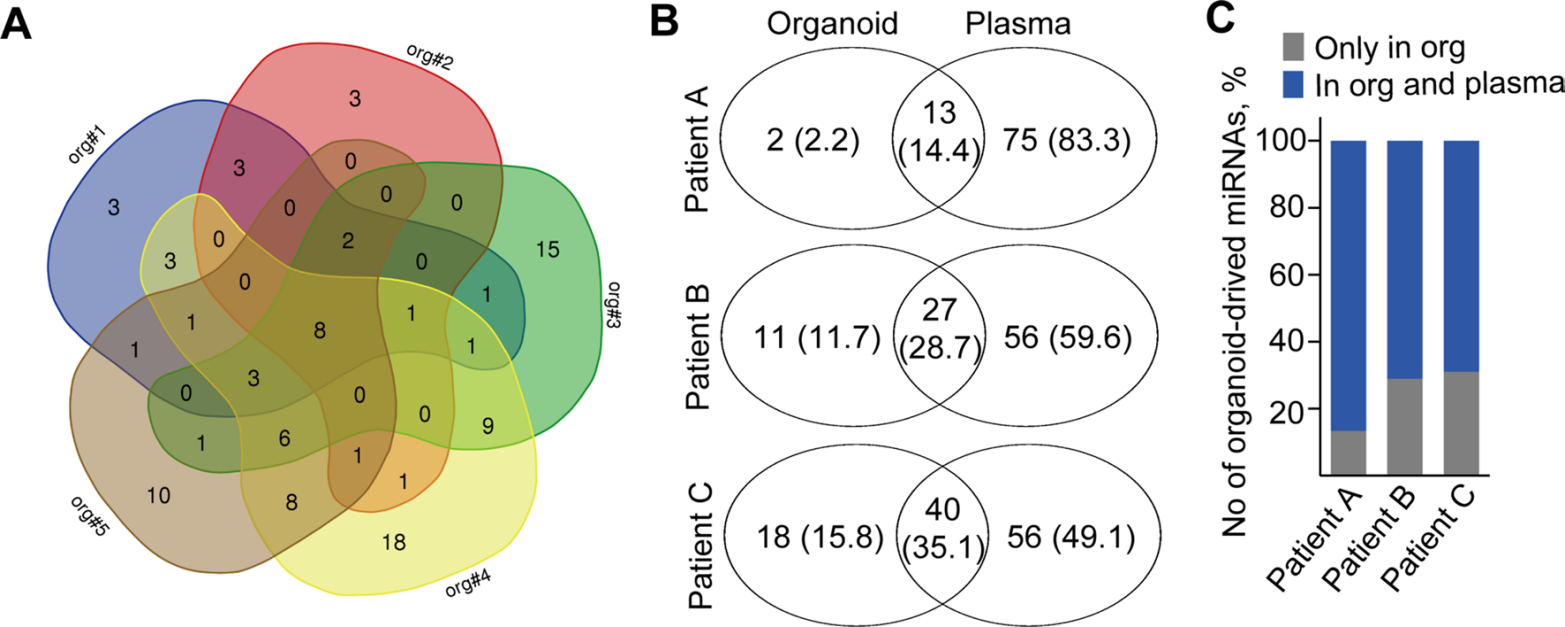
Screen of EV-miRNAs derived from five human PDAC organoid lines shows a common miRNA set. **a** Venn-diagram of the number of miRNAs detected in PDAC organoid lines. Note that a Ct value < 42 was regarded as positive hit. Supernatants were isolated from cultures with identical cell numbers. **b**–**c** Comparison of PDAC organoid-derived EV-miR profiles with plasma EVs of three individual patients. Data for the individual patients are shown in (**b**). Note that the majority of organoid-derived EV miRNAs are present in the plasma EVs as well (**c**). Numbers in parenthesis indicate the percentage of miRNAs detected in organoid and/or plasma EVs

We then carried out miRNA profiling of EVs from five patient-derived organoid lines (Table S6). To ensure that we isolate as many EVs as possible, we used a mixture of anti-CD63 and anti-CD81-coated beads. Importantly, miRNAs detected in Matrigel-derived supernatant without cells (control sample) were removed from further data analysis. Interestingly, when analyzing EVs according to their presence (Ct < 42) or absence in the samples, we found a set of miRNAs that were present in EVs derived from the individual patient-derived organoid lines and eight miRNAs overlapped among all the cultures (Fig. 5a).

We next carried out a pairwise comparison of the miRNA profiles of EVs derived from PDAC organoids with the cargo of the corresponding blood plasma EVs, isolated by anti-CD81 and anti-CD63-coated beads. Importantly, samples (organoids and plasma) were derived from the same patient (Table S6). Since organoid culture supernatants and blood plasma represent different sample types, where the direct comparison of Ct values is not informative, we decided to score miRNAs according to their presence (Ct < 42) or absence in the samples. miRNAs present in the Matrigel control were removed from the analysis. We detected a higher number of plasma-derived EV-miRs compared to the number of EV-miRs in PDAC organoid supernatant from the same patient (Fig. 5b). In addition, 75.6 + 9.7% (mean + SD) of the miRNAs present in organoid EVs were found in the corresponding PDAC blood EVs as well (Fig. 5c and Tables S7–S8), highlighting the presence of a large percentage of PDAC tumor-cell produced EV-miRs in the systemic circulation. Furthermore, when listing miRNAs common in PDAC organoid and blood-derived EVs in individual pairwise comparisons, the overlapping set of the comparisons resulted in eight miRNAs that we called “PDAC organoid common set” (POCS) (Table 2). Thus, these results show that the vast majority of PDAC organoid EV miRNAs are present in the blood samples as well. Furthermore, they highlight the large variance among patients. Importantly, the POCS miRNA set completely overlapped with the common miRNAs derived from the supernatants of all five organoid lines; however, not all of them were present in the commercially available PDAC cell line-derived EVs (Table 2).

**Table 2:** POCS miRNA set, present in all PDAC organoid EVs, shows a complete overlap with PDAC blood EVs and a partial overlap with PDAC cell line (Panc10.05, Panc08.13)-derived EV cargo (cultured in Matrigel)

POCS	Presence in EVs in …		
	All PDAC organoid supernatants	All PDAC cell line supernatants	All PDAC plasma samples
hsa-miR-146a	✓	Only in Panc08.13	✓
hsa-miR-16	✓	✓	✓
hsa-miR-24	✓	✓	✓
hsa-miR-320	✓	Only in Panc08.13	✓
hsa-miR-192	✓	Only in Panc10.05	✓
hsa-miR-222	✓	✓	✓
hsa-miR-21	✓	✓	✓
hsa-miR-19b	✓	✓	✓

### Both PDAC and CP patients have an elevated number of CD63 + EVs in the blood plasma

**Fig. 6:**
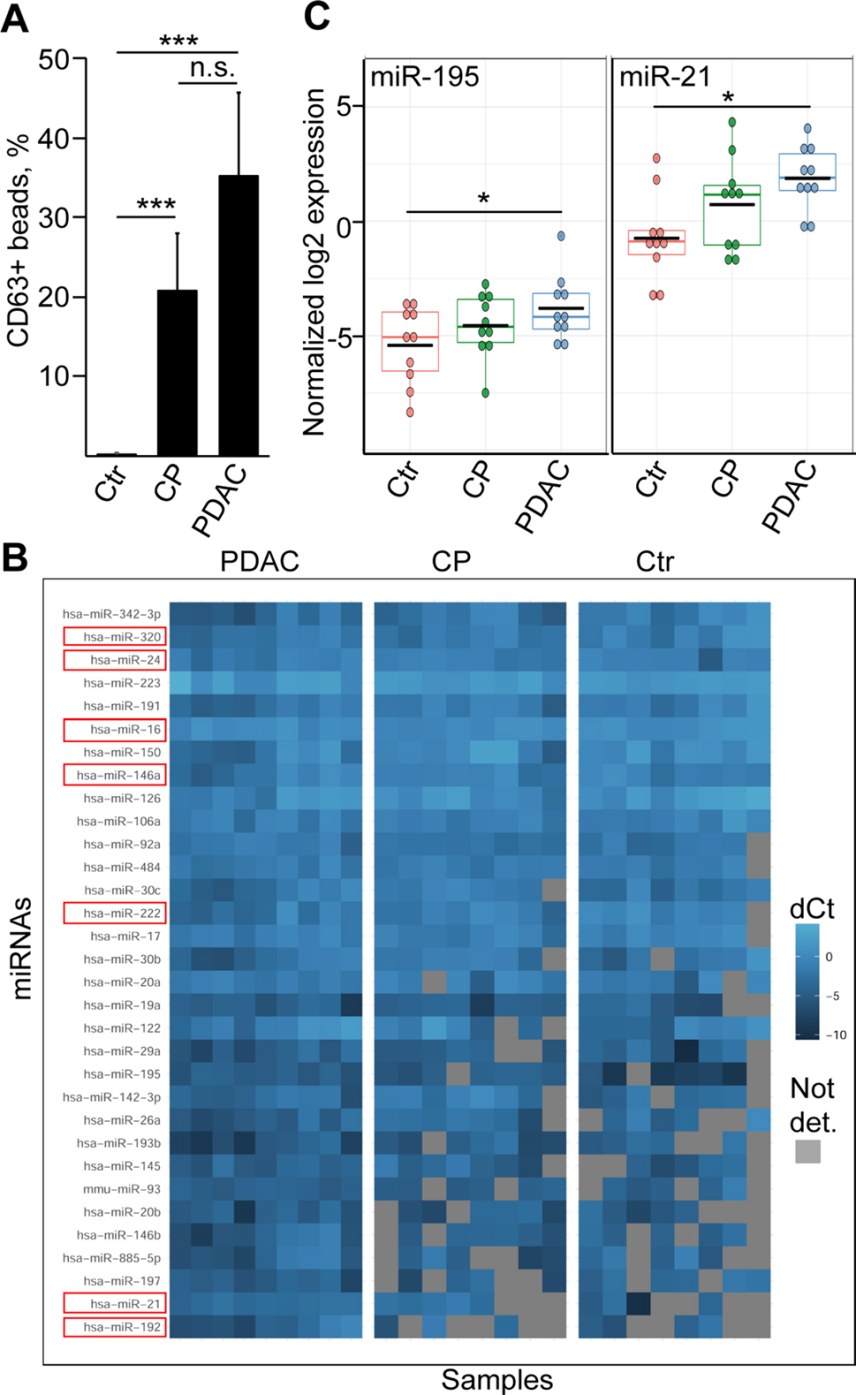
miRNAs present at an elevated level in PDAC blood-derived EVs are not specific for this disease. **a** The percentage of CD63 + beads after incubating them in the plasma samples from the indicated patient groups, detected by flow cytometry (*n* = 8–11). *CP* chronic pancreatitis, *Ctr* healthy control subjects. Note that identical volume of blood was applied in the experiments. **b** Comparison of the normalized levels of miRNAs present in all PDAC plasma EV samples (PDAC) to miRNA levels of CP and control subjects. Grey squares depict miRNAs that were absent in the samples. miRNAs that were present in the POCS set are marked with a red color. Note that since data were normalized to miR-19b, this miRNA is not shown on the graph. **c** Normalized miR-21 and miR-195 levels of the EV preparates. ANOVA and Tukey’s post hoc test (**a**) or Kruskal–Wallis and Dunn test (**c**) were used. **p < 0.01, ***p < 0.005

Since the access to normal human pancreas tissue is limited, determining the PDAC-specific subset of POCS is difficult. However, these miRNAs were present in PDAC blood plasma EVs as well. Thus, we compared PDAC to CP patient-derived and normal blood plasma EVs. First, we examined the differences in the amounts of EVs in samples derived from the plasma of PDAC or CP patients or control donors. Interestingly, when anti-CD63-coated beads were incubated in the plasma that had been centrifuged to remove larger EVs and EVs bound to beads were detected by anti-CD63 and flow cytometry, we observed an increased percentage of positive beads for CP and PDAC samples compared to controls. In contrast, no significant difference was found between the CP and the PDAC groups (Fig. 6a). Importantly, EVs captured by anti-CD63-coated beads could be detected with anti-CD63, but not with anti-CD81, suggesting that these EVs were positive only for CD63 **(**Fig. S3A). In addition, the percentage of positive beads dropped when they were incubated in the supernantant after ultracentrifugation that is EV-free or when adding Triton X-100 after the detection antibody (Fig. S3A, B). These results prove that when using anti-CD63-coated beads and anti-CD63 detecting antibody, we measure EVs and not free CD63 proteins in the plasma. Collectively, the elevated number of CD63 + EVs, detected by the quick and semi-quantitative bead-based method and flow cytometry, is not specific for PDAC.

### PDAC and CP patient blood-derived EVs have a largely overlapping miRNA profile

We next compared miRNA profiles of EVs isolated by anti-CD63 and anti-CD81-coated beads from the blood of PDAC, CP patients and controls using low-density miRNA arrays (Table S6). To rule out differences in the miRNA content due to variations in EV amount of the starting material, miRNA levels were normalized to miR-19b. We selected miRNAs present in all PDAC plasma samples and compared their normalized levels to CPs and controls. Surprisingly, all POCS-miRNAs were present not only in PDAC plasma EV samples, but in CPs and controls as well (Fig. 6b). Interestingly, we detected two miRNAs that were significantly enriched in PDAC plasma EV preparations (miR-195 and miR-21) as compared to control samples (Fig. 6c). However, none of them differed when analyzing PDAC and CP blood EVs (Benjamini–Hochberg FDR, *p* < 0.05) (Table S6). Collectively, when using a complex approach with blood samples and PDAC organoids, we identified a set of EV miRNAs characteristic for PDAC organoids, which were detected in PDAC patient plasma-derived EVs as well. However, the EV miRs that showed a difference between PDAC and control blood samples did not differ between PDAC and CP.

### miR-195 is not detected in EVs from PDAC tumor cells or fibroblasts

Using individual miRNA assays, we then confirmed our results for miR-195 and miR-21 from plasma-derived EVs (normalized to miR-19b) (Fig. 6c). Next we wanted to see whether EVs enriched for miR-195 and miR-21 could originate from PDAC epithelial cells and/or from stromal cells. To accomplish this, we cultured PDAC tumor-derived stromal cells. As expected, these cells showed a fibroblastic phenotype and we confirmed the expression of markers of cancer-associated fibroblast subtypes (Fig. S4A) [24]. In line with our previous results, PDAC cell line and PDAC organoid-derived EVs contained miR-21 abundantly, but had an undetectable level of miR195 (Fig. S4B). Similarly, miR-195 was not detected in stromal fibroblasts either (Fig. S4B).

### Collagen deposition induces EV release from pancreatic ductal organoids

PDAC cell lines produced a higher amount of EVs compared to the control immortalized ductal cell line and not only PDAC, but CP patient blood plasma samples had an elevated level of EVs. These raised the possibility that not only mutations, but other factors may result in an enhanced EV release from pancreatic ductal cells. To test this hypothesis, we established a mouse pancreatic ductal organoid model that represents a genetically homogenous model system. As expected, organoids expressed the ductal markers *Krt19*, *Pdx1*, *Sox9*, but the acinar and endocrine markers *Cpa1*, *Ptf1a, Ins2* and *Chga* were only hardly or not detected by RT-qPCR (Fig. S5A). Importantly, TEM images proved the presence of EVs in organoid supernatants (Fig. S5B).

**Fig. 7:**
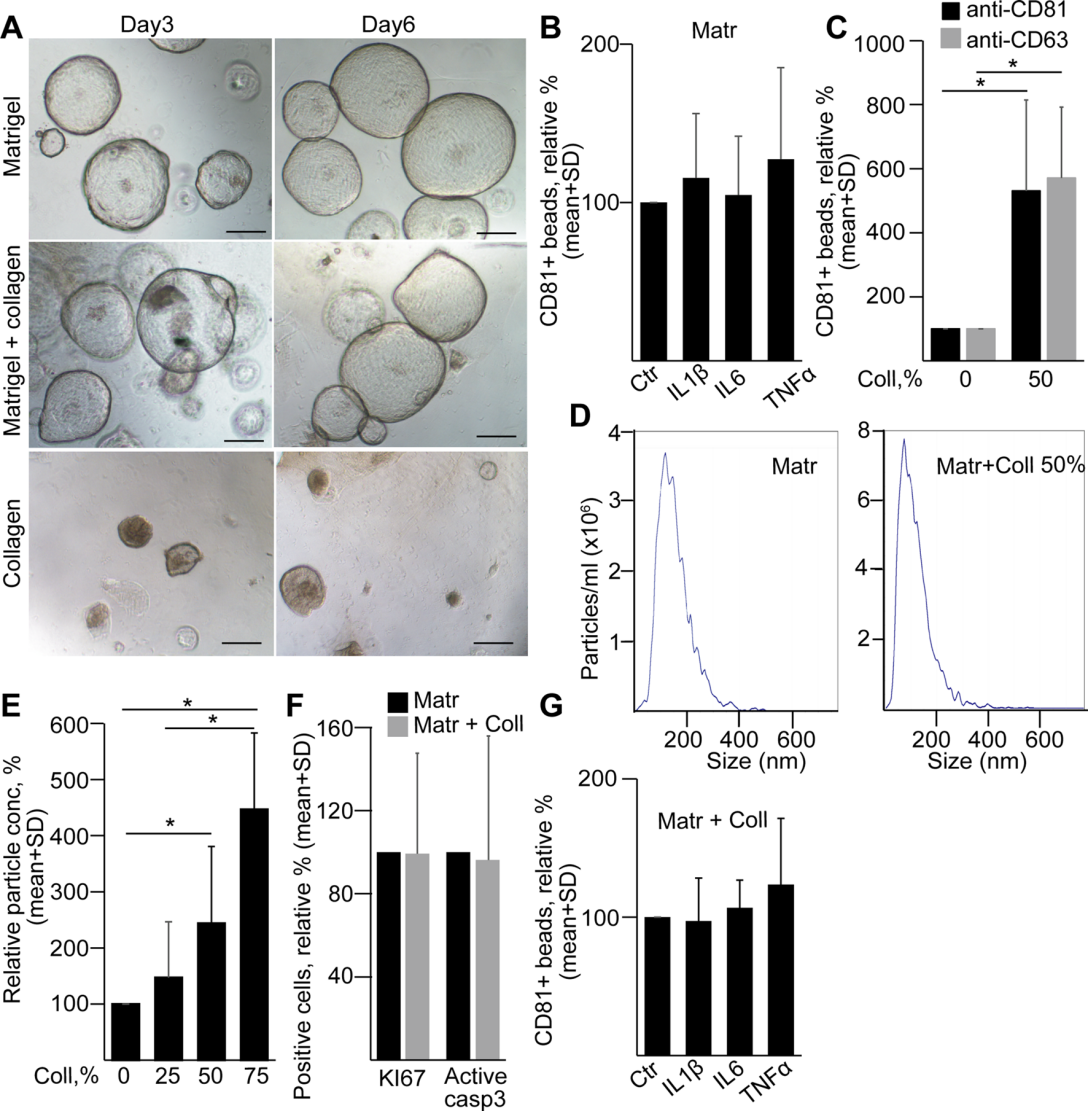
Collagen I induces EV release from normal mouse pancreatic ductal organoids. **a** The morphology of organoids grown in Matrigel, Matrigel and collagen I (50–50%) or in collagen I 3 and 6 days after splitting. Scale bars: 100 µm. **b** The relative percentage of CD81 + beads after the indicated treatments (*n* = 4–5). Cultures were treated for 3 days before collecting EVs for 2 days. **c** CD81 + beads from organoids cultured in Matrigel with no collagen (0%) or 50% collagen I (*n* = 5). Positive beads were detected by either anti-CD81 or anti-CD63. **d**–**e** NTA representative images (**d**) and relative particle concentrations (**e**), measured from organoid supernatants at different collagen contents (*n* = 7). Data were normalized to cell number and then the Matrigel control was always taken as 100%. **f** The relative percentage of the KI67 + proliferating and active caspase-3 + apoptotic organoid cells in Matrigel without or with 50% collagen I (*n* = 3). **g** The relative percentage of CD81 + beads (detected by anti-CD81) from organoids cultured in Matrigel or 50% Matrigel/50% collagen (*n* = 5). Paired *t* test (**b**, **c**, **f** and **g**) or one-way ANOVA and Tukey post hoc test (**e**) were used. *p < 0.05

A recent comprehensive study identified that many microenvironmental factors, including changes in the PDAC extracellular matrix (ECM), such as the accumulation of collagen, are already present in CP [25]. Thus, we focused on factors with known role in CP, such as collagen, IL-6, TNFα and IL-1β [26, 27]. Interestingly, wild type pancreatic ductal organoids had an extensive cell death when they were cultured in pure collagen I (Fig. 7a). We found no effect of IL-6, TNFα, or IL-1β on EV release (Fig. 7b). However, the addition of collagen I to Matrigel increased EV secretion from ductal organoids, detected when EVs were isolated with anti-CD81-coated beads and measured with either anti-CD81 or anti-CD63 (Fig. 7c). In addition, we found a correlation between the increasing collagen concentration and EV number by NTA (Fig. 7d, e). Importantly, normalization of data to cell numbers excluded differencies in the detected EV amounts due to variations in cell numbers. Furthermore, we found no significant change in the percentage of active caspase-3 + apoptotic cells and proliferating KI67 + cells when adding 50% collagen to Matrigel (Fig. 7f). In addition, IL-6, TNFα or IL-1β did not result in a massive change of EV release when organoids were cultured in collagen/Matrigel either (Fig. 7g). Collectively, these results provide evidence that not only mutations, but also critical changes in the cellular microenvironment can lead to a striking increase in EV production in pancreatic ductal cells. This may explain the increased amount of CD63 + EVs detected not only in PDAC, but also in CP patient-derived blood samples.

## Discussion

In this study we used a complex approach to detect EV miRNAs characteristic for PDAC patients. Interestingly, only a small subset of the miRNAs overlapped when we compared EVs derived from different organoids, showing a high individual variance among patients. However, EV miRNAs common in all organoids were present in PDAC patient-derived blood EVs as well. In addition, we identified two miRNAs (miR-21 and miR-195) that were present in a higher amount in PDAC plasma EV preparations than in controls, albeit we found no difference compared to CP samples. To exclude miRNA changes due to alterations in the EV content among samples, we used miR-19b as a normalization control, a miRNA showing the highest stability among our samples and absent in all control media. Furthermore, immune affinity capture isolation of EVs by anti-CD81 or anti-CD63-coated beads in our studies ensured that the detected miRNAs were indeed encapsulated in EVs rather than being associated with Ago2 or HDL, two known other carriers of extracellular RNAs. In this study, we also identified collagen deposition as a critical factor inducing EV release from pancreatic ductal cells. Since the accumulation of collagen in the extracellular matrix (ECM) is characteristic for both CP and PDAC, this provides a possible explanation to our observation that both CP and PDAC patient-derived plasma samples have an elevated amount of CD63 + EVs.

Since EVs are considered as potential tools for PDAC diagnostics, many studies have already analyzed the cargo of EVs using cell lines and led to different outcomes. One possible explanation for the discrepancies may be the varying culture conditions and EV isolation methods that are known to largely affect which molecules are detected [28]. EV cargo from 2 and 3D cultures have already been compared for gastric cancer [29] and cervical cancer cells [30] and the authors found that EVs from 2 and 3D cultures differed in some of their miRNAs. Interestingly, here we found that the 3D matrix is critical for the miRNA content of EVs. Whereas Matrigel had only a minor effect on the cargo compared to EVs derived from 2D cultured cells, the addition of collagen resulted in a change of EV miRNA levels and in a modified phenotype of 3D spheroids. Importantly, unlike other studies that mostly used ultracentrifugation, we detected miRNAs from EVs isolated by the antibody-coated bead-based method, an approach that we found earlier to result in a lower unspecific background [21]. Thus, similarly to others, we concluded that the 3D culture system is a more useful model for mimicking in vivo environment in studying EV release and functions than the 2D one.

The strength of patient-derived organoids is that they represent the genetic and cellular heterogeneity characteristic for in vivo tumors [31]. We previously proved that organoids represent a suitable model to study EV release and cargo in colorectal cancer [21]. To our knowledge, this is the first study using PDAC patient-derived organoids for EV analysis. Despite the high variation among the individual patient-derived EVs, we identified a set of overlapping miRNAs. Since organoids maintain the cellular heterogeneity of the tissue of origin, the presence of different cancer cell subpopulations may critically determine the cargo of EVs, resulting in the large variation among patients.

By identifying patients already at an early stage of the disease, Melo S et al. found that EV-bound glypican-1 is a specific biomarker for PDAC [32]. However, other studies came to other conclusions [33, 34], potentially because of using other EV isolation methods or cargo analysis criteria. Upregulation of circulating miRNAs in plasma and serum of PDAC patients has been shown for several miRNAs [34, 35]. Other publications suggested HULC lncRNA [36], a combination of eight EV long RNAs [37] or a signature of five protein serum markers [38] as potential biomarkers. Furthermore, Ko J et al. identified a biomarker panel of eleven EV miRNAs that distinguished mice with PDAC from either healthy animals or mice with precancerous lesions [39]. In contrast to other reports, we used anti-CD63 and anti-CD81-coated beads. Surprisingly, miR-21 and miR-195 were present in a higher amount in PDAC EV preparates than in controls, albeit we found no difference when compared to CP samples. This clearly highlights the strong need to include CP samples into biomarker research when looking for pancreatic cancer EV biomarkers. High levels of miR-21 may have an oncogenic potential in lung cancer, while low levels are required for differentiation and development. Thus, its level is critical for balancing cellular proliferation and differentiation [40]. The role of miR-195 has been studied intensively, and its dysregulation has been reported in several cancer types [41]. It has been recognized as a circulating miRNA and its normalized serum levels have been suggested as a serological biomarker in other cancers [42].

Importantly, EVs with cargo characteristic for PDAC blood plasma can be secreted not only by PDAC tumor cells, but also by other cells such as stromal cells of the tumor tissue. Since organoids contain only cells of epithelial origin, e.g., tumor cells, but not stromal cells, they provide an excellent model to decide which EV miRNAs of the blood are not of cancer cell origin. This application of the organoids is further strengthened by our observation that organoid and blood plasma-derived EVs have a largely overlapping miRNA profile in the same patient. Interestingly, whereas miR-21 was present in both PDAC organoid-derived and blood EVs, we could not detect miR-195 in the organoid or PDAC cell line-derived EVs. This may suggest that circulating EVs with miR-195 are not derived from tumor cells. This notion is also supported by Li et al. who detected miR-195 in EVs secreted by cancer-associated fibroblast (CAF) in cholangiocarcinoma [43]. Similarly, Zhou et al. found a low level of this miRNA in primary PDAC cells compared to controls [44]. However, we could not detect an increased amount of miR-195 in fibroblast-derived EVs either, suggesting that in our experimental system miR-195 encapsulated in EVs is not of tumor cell or stromal fibroblast origin.

Using organoids, we identified the accumulation of collagen type I as a possible factor inducing EV release from pancreatic ductal cells. This may lead to the higher CD63 + blood plasma EV levels not only in PDAC, but in CP patients as well. Similarly to PDAC, CP is mostly accompanied by desmoplasia and by ECM remodeling with the increase in fibronectin, collagen type I and V [45]. In addition, a recent analysis identified widespread changes in the ECM in CP [25]. In line with our results, a recent report showed that CD43 + cells released EVs in higher amounts when cultured on collagen type I biomaterial. Moreover, the authors found elevated levels of miR-21 and miR-201 in these EVs [46]. Although a large-scale screen of microenvironmental factors inducing EV secretion from pancreatic ductal cells is still lacking, our results suggest that the ECM has a critical effect on EV release independently of tumor-specific driver mutations already at a pre-tumorigenic stage.

Collectively, we found that miR-21 and miR-195 are present in elevated amounts in PDAC blood plasma EV samples compared to controls, albeit these miRNAs are not specific for PDAC. Using organoids, we showed the overlap between the miRNA cargo of PDAC cell-derived and circulating EVs. Interestingly, our results suggest that EV miR-195 in the plasma is not of tumor origin. In addition, our data showed that the accumulation of collagen type I in the ECM substantially induced EV release from pancreatic ductal cells, providing a possible explanation for the elevated EV levels in both CP and PDAC blood samples. Taken together, our data suggest that patient-derived organoids are a highly relevant tool when dissecting the cargo and cellular origin of EVs in biomarker research. Furthermore, we provide evidence that not only mutations, but also changes in the ECM may critically modify EV release from pancreatic ductal cells.

### Electronic supplementary material

Below is the link to the electronic supplementary material.

Supplementary file1 (PDF 2032 kb)

Supplementary file2 (XLSX 51 kb)

Supplementary file3 (XLSX 98 kb)

## Data Availability

Data are available from the corresponding author upon reasonable request.
