# Supplementary material for: Shared extracellular vesicle miRNA profiles of matched ductal pancreatic adenocarcinoma organoids and blood plasma samples show the power of organoid technology
Source: Cell Mol Life Sci. 2020 Nov 25;78(6):3005–20. doi: 10.1007/s00018-020-03703-8 (PMC8004523; doi:10.1007/s00018-020-03703-8)
Supplement: Supplementary file 1 — Supplementary file1 (PDF 2032 kb) [file 18_2020_3703_MOESM1_ESM.pdf]

Cellular and Molecular Life Sciences

Shared extracellular vesicle miRNA profiles of matched ductal pancreatic adenocarcinoma organoids and blood plasma samples show the power of organoid technology

Anikó Zeöld*, Gyöngyvér Orsolya Sándor*, András Áron Soós, Anna Kiss, Tamás Tölgyes, Attila Bursics, Ákos Szűcs, László Harsányi, Ágnes Kittel, András Gézsi, Edit I Buzás, Zoltán Wiener[&]

*shared authorship

[&]corresponding author: Zoltán Wiener, Semmelweis University, Budapest, Hungary, e-mail: wiener.zoltan@med.semmelweis-univ.hu

	TLDA sample names		PFP qPCR	Gender	Age
	PFP (plasma)	organoid supnat			
PDAC	-	org1	-	f	52
	PDAC8 (Patient B)	org2	yes	f	63
	-	org3	-	f	68
	PDAC7 (Patient A)	org4	yes	m	48
	PDAC9 (Patient C)	org5	yes	f	77
	PDAC 1	-	yes	f	77
	PDAC 2	-	yes	f	72
	PDAC 3	-	yes	f	68
	PDAC 4	-	yes	f	68
	PDAC 5	-	yes	m	71
	PDAC 6	-	yes	m	67
	-	-	yes	m	59
Inflammation	CP1		yes	f	66
	CP2		yes	m	65
	CP3		yes	f	36
	CP4		yes	f	65

	CP5		yes	m	49
	CP6		yes	m	67
	CP7		yes	f	60
	-		yes	m	56
	CP8		yes	f	61
	-		yes	f	80
ctrl	ctrl1	-	yes	f	57
	ctrl2	-	yes	f	50
	ctrl 3	-	yes	f	66
	ctrl 4	-	yes	f	63
	ctrl 5	-	yes	m	58
	ctrl 6	-	yes	m	55
	ctrl7	-	yes	f	54
	ctrl8	-	yes	m	44
	-	-	yes	f	45
	-	-	yes	m	54

Table S1. Clinical parameters of the patients/subjects involved in our studies.

**Antibody**	**Source**	**Clone/Cat No**
FITC anti-human CD81	Molecular Probes	A15753
PE anti-human CD63	Sigma	SAB4700218
Anti-CD81 antibody	Thermo Scientific	MA180209
anti-human CD81	Sigma	SAB3500454
PE anti-CD81	Thermo Fisher	MA517941
anti-active Caspase3	R&D Systems	AF835
anti-KI67	Abcam	ab16667
anti-rabbit IgG Alexa 568	Invitrogen	A11011
anti-rabbit IgG Alexa 488	Invitrogen	A21206
anti-mouse IgG Alexa 488	Invitrogen	A21202
anti-IL6	Abcam	ab214429
anti-αSMA	Merck-Sigma	A5228

Table S2. Antibodies used in our experiments.

**Primer name**	**Sequence**
hKRT19	Hs00761767_s1
hPDX1	Hs00236830_m1
hSOX9	Hs001165814_m1
hPTF1A	Hs00603586_g1
hCPA1	Hs01056157_m1
hCHGA	Hs00900370_m1
hINS	Hs00355773_m1
hHPRT	Hs02800695_m1
hGAPDH	Hs02786624_g1
mKrt19	Mm00492980_m1
mPdx1	Mm00435565_m1
mSox9	Mm00448840_m1
mChga	Mm00514341_m1
mCpa1	Mm00465942_m1
mIns2	Mm00731595_g1
mPtf1	Mm00479622_m1
mHprt1	Mm03024075_m1
mGapdh	Mm99999915_g1
hsa-miR128-3p	477892_mir
hsa-miR181a-5p	477857_mir
hsa-miR181b-5p	478583_mir
hsa-miR222-3p	477982_mir
hsa-miR484	478308_mir
hsa-miR21-5p	477975_mir
hsa-miR195-5p	477957_mir
hsa-miR19b-3p	478264_mir

Table S3. TaqMan assays and advanced TaqMan miR assays used in our studies.

Table S4. miRNA proofile of EVs isolated from PDAC cells lines cultured 2D or 3D in different matrices.

Organoid	Survival w/o EGF and w/o noggin	KRAS	TP53	Survival with nutlin-3
org #1	**yes**	3' UTR	S96P	yes
org #2	**yes**	wt	R248Q	yes
org #3	**yes**	n.d.	n.d.	yes
org #4	**yes**	G12V	R249S	yes
org #5	**yes**	wt	K132N, V173E	yes

Table S5. Features of the PDAC organoids.

Table S6. miRNA data from PDAC organoid, PDAC, CP and control blood-derived EVs (separate Excel sheet).

% of organoid derived EV miRs present in blood EVs as well	
patient A	86.7
patient B	71.1
patient C	69.0
mean	75.6
SD	9.7

Table S7. The percentage of PDAC organoid EV miRNAs that are present also in the plasma EVs from the same patient.

Patient A	Patient B		Patient C		
hsa-miR-16	hsa-miR-9	hsa-miR-200c	hsa-let-7g	hsa-miR-146a	hsa-let-7b
hsa-miR-19b	hsa-miR-16	hsa-miR-215	hsa-miR-16	hsa-miR-146b	hsa-miR-365
hsa-miR-20b	hsa-miR-17	hsa-miR-222	hsa-miR-17	hsa-miR-181a	hsa-miR-374
hsa-miR-21	hsa-miR-19b	hsa-miR-223	hsa-miR-19a	RNU48	mmu-miR-374-5p
hsa-miR-24	hsa-miR-21	hsa-miR-224	hsa-miR-19b	hsa-miR-186	hsa-miR-375
hsa-miR-29a	hsa-miR-24	hsa-miR-320	hsa-miR-20a	hsa-miR-192	hsa-miR-422a
hsa-miR-30b	hsa-miR-92a	hsa-miR-323-3p	hsa-miR-21	hsa-miR-193b	hsa-miR-429
hsa-miR-146a	hsa-miR-106b	hsa-miR-345	hsa-miR-24	hsa-miR-194	hsa-miR-485-3p
hsa-miR-192	hsa-miR-126	hsa-miR-372	hsa-miR-27b	hsa-miR-197	hsa-miR-487a
hsa-miR-193b	hsa-miR-133b	hsa-miR-375	hsa-miR-29a	hsa-miR-198	hsa-miR-509-5p
hsa-miR-200c	hsa-miR-141	hsa-miR-422a	hsa-miR-30c	hsa-miR-199a-3p	hsa-miR-518b
hsa-miR-222	hsa-miR-145	hsa-miR-489	hsa-miR-31	hsa-miR-200b	hsa-miR-518d
hsa-miR-302a	hsa-miR-146a	hsa-miR-518d	hsa-miR-34a	hsa-miR-222	hsa-miR-532
hsa-miR-320	hsa-miR-181a	hsa-miR-522	hsa-miR-92a	hsa-miR-223	hsa-miR-548c
hsa-miR-590-5p	RNU48	hsa-miR-532	hsa-miR-95	hsa-miR-320	hsa-miR-597
	hsa-miR-190	hsa-miR-574-3p	hsa-miR-106b	hsa-miR-323-3p	hsa-miR-627
	hsa-miR-192	hsa-miR-597	hsa-miR-126	hsa-miR-324-3p	hsa-miR-636
	hsa-miR-194	hsa-miR-636	hsa-miR-132	hsa-miR-331	hsa-miR-708
	hsa-miR-200b	hsa-miR-885-5p	hsa-miR-141	hsa-miR-335	hsa-miR-325
			hsa-miR-145		

Table S8. List of the detected miRNAs in PDAC organoid EVs. miRNAs present in the blood EVs in the same patient are highlighted.

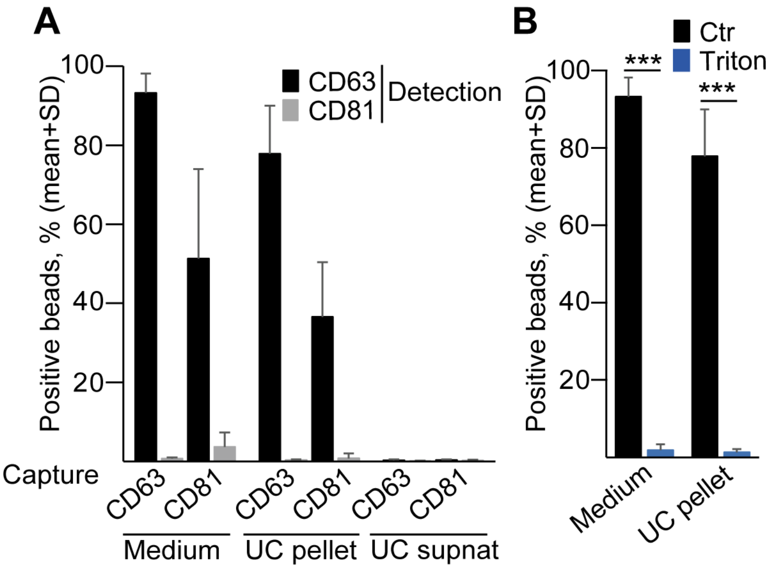

Figure S1. PDAC cell line-derived EVs accumulate in the pellet after ultracentrifugation. A) The percentage of anti-CD63 and anti-CD81-coated beads after incubating them in Panc10.05 PDAC cell line-derived medium, in the pellet or in the supernatant after ultracentrifugation and the positive beads were detected by anti-CD63 or anti-CD81 (flow cytometry, n=4). B) The percentage of anti-CD63 coated positive beads after detection with anti-CD63 and before (Ctr) or after incubation with 0.05% Triton X-100 (Triton) (n=4). t-test was used (B) with ***p<0.005

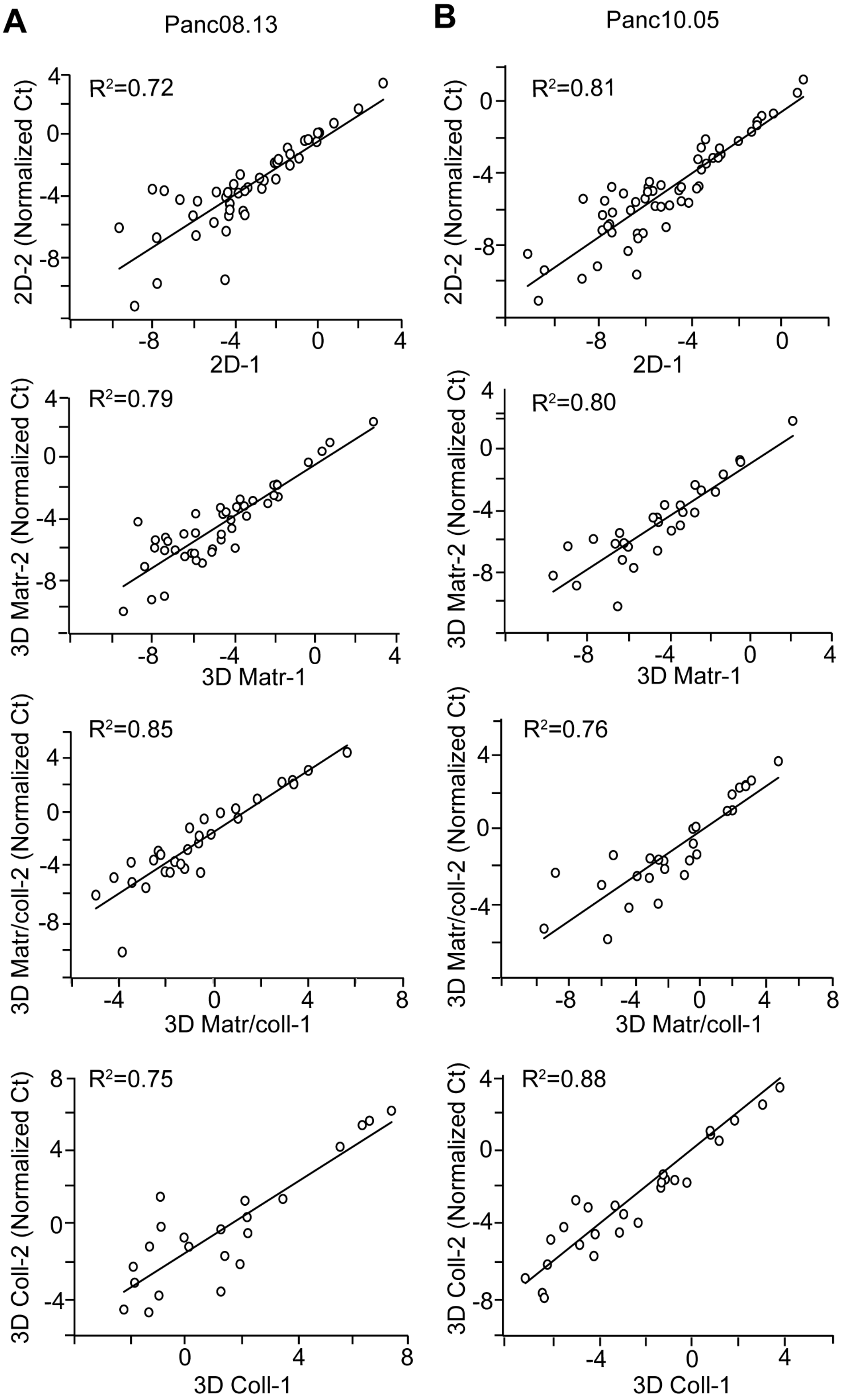

Figure S2. Replicates of EV miRNA screens show a high correlation. A-B) Correlation of EV miRNA levels between the two replicates for Panc08.13 (A) and Panc10.05 (B). EVs were collected from cells cultured 2D or 3D in the indicated matrices (low density miRNA card). Ct values were normalized to the level of miR-19b.

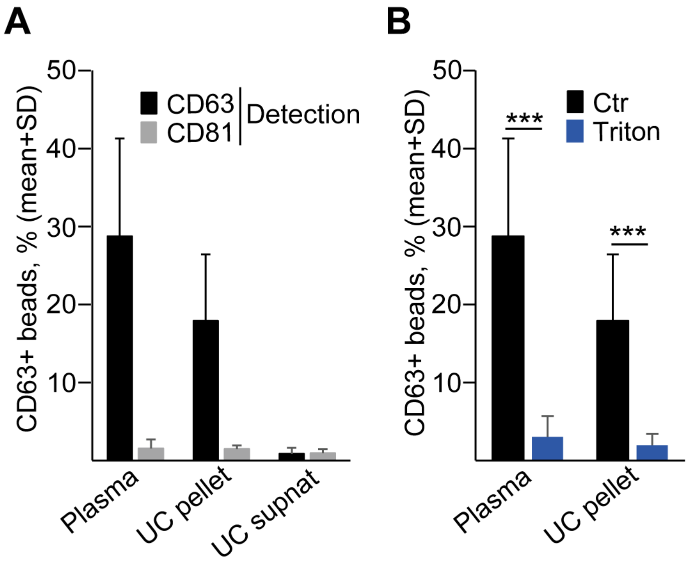

Figure S3. PDAC plasma EVs can be detected by anti-CD63-coated beads and anti-CD63. A) The percentage of positive anti-CD63 coated beads after incubation in PDAC blood plasma or in the pellet/supernatant of ultracentrifuged plasma samples and detected by anti-CD63 or anti-CD81 (flow cytometry, n=4). B) Anti-CD63 coated positive bead percentage after detection with anti-CD63 and before (Ctr) or after incubation with 0.05% Triton X-100 (Triton) (n=4). t-test (B) with ***p<0.005.

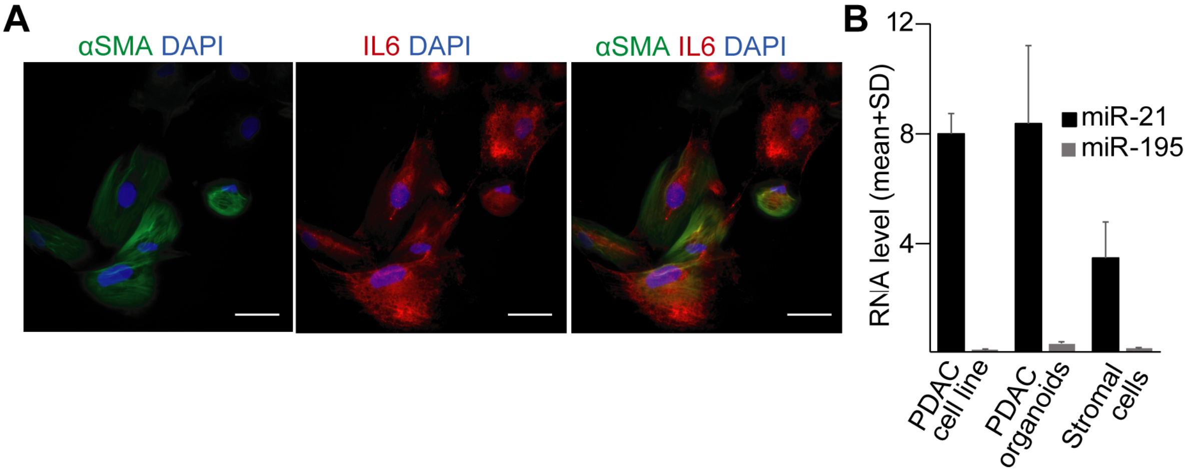

Figure S4. Both PDAC tumor cell and stromal cell-derived EVs contain miR-21, but not miR-195. A) Immunostaining of cell cultures derived from PDAC samples for markers of different fibroblast subpopulations (IL-6, αSMA). Scale bar: 10µm. B) miR-21 and miR-195 levels from EV preparates isolated from Panc10.05 cells, PDAC organoids or stromal fibroblasts (n=5).

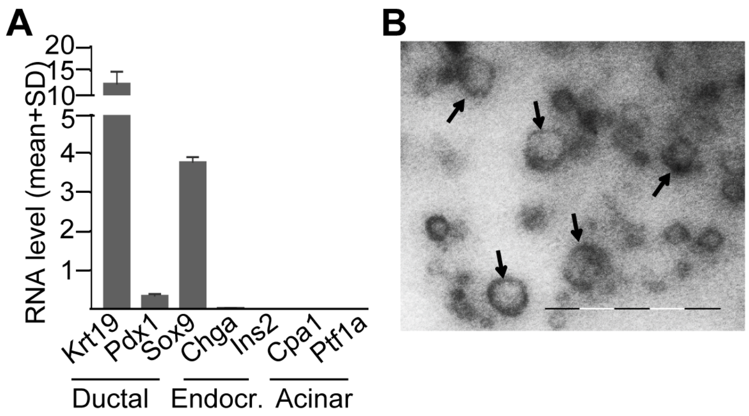

Figure S5. Mouse pancreas-derived organoids express ductal markers. A) Relative RNA level of ductal, endocrine and acinar markers (RT-qPCR). B) Detecting EVs (arrows) in mouse pancreas organoid-derived supernatant with transmission electron microscopy (TEM). Scale bar: 500 nm (C).
